# Lethal Means Counseling for Suicidal Adults in the Emergency Department: A Qualitative Study

**DOI:** 10.5811/westjem.2021.8.49485

**Published:** 2021-05-07

**Authors:** Bonnie J. Siry, Christopher E. Knoepke, Stephanie M. Ernestus, Daniel D. Matlock, Marian E. Betz

**Affiliations:** * University of Colorado School of Medicine, Department of Emergency Medicine, Aurora, Colorado; † University of Colorado School of Medicine, Division of Cardiology, Aurora, Colorado; ‡ University of Colorado School of Medicine, Adult & Child Consortium for Outcomes Research & Delivery Science, Aurora, Colorado; § University of Southern California, USC Suzanne Dworak-Peck School of Social Work, Los Angeles, California; ¶ Stonehill College, Department of Psychology, Easton, Massachusetts; || University of Colorado School of Medicine, Department of Geriatric Medicine, Aurora, Colorado; # VA Eastern Colorado Geriatric Research Education and Clinical Center, Aurora, Colorado

## Abstract

**Introduction:**

Lethal means counseling (to reduce access to firearms or other suicide methods) is a recommended critical yet challenging component of care of suicidal patients. Questions remain about communication strategies for those in acute crisis.

**Methods:**

This qualitative study was an analysis of semi-structured interviews with English-speaking, community-dwelling adults with a history of lived-experience of suicidal ideation or attempts in themselves or a family member. We used a mixed inductive and deductive approach to identify descriptive themes related to communication and decision-making.

**Results:**

Among 27 participants, 14 (52%) had personal and 23 (85%) had family experience with suicide ideation or attempts. Emergent themes fell into two domains: (1) communication in a state of high emotionality; and (2) specific challenges in communication: initiating, maintaining engagement, considering context.

**Conclusion:**

Engaging suicidal individuals in lethal means counseling may be more effective when messaging and approaches consider their emotional state and communication challenges.

## INTRODUCTION

Emergency departments (ED) are the site where patients with acute suicidal ideation or attempts (SI/SA) are generally sent for immediate evaluation and intervention. There is a spectrum of interventions for patients with SI/SA, from inpatient psychiatric hospitalization to outpatient follow-up. Lethal means counseling (LMC) – counseling meant to reduce access to firearms, medications, and other highly lethal methods is recognized as an essential, evidence-based component of suicide prevention,^1^ especially for patients being discharged home. Prior work has shown that LMC may positively affect home storage behaviors, especially among parents of suicidal adolescents.^2^,^3^

Yet LMC in the ED does not routinely occur with suicidal adults. Even among those being discharged home, counseling is documented in only about half of these patients.^4^ Identified barriers to counseling include unclear provider responsibilities (e.g., whether ED or behavioral health clinicians should provide counseling^5^,^6^), lack of protocols or training (for both ED and behavioral health clinicians), and hesitancy about discussing firearms with patients.^7^ In response, organizations have called for increased clinician training and engagement in LMC,^8^,^9^ highlighting the need for identifying evidence-based best messages and messengers for this work.^10^,^11^ As an example, “means safety” (vs “means restriction”) was both more acceptable to participants and made participants more willing to consider reducing access to lethal means.^12^ Other evidenced-based work underscored the need for engaging the firearms community in developing “culturally specific” messaging, such as drawing on the values of safety, responsible ownership, and protection of loved ones.^13^,^14^

While efficacy and clinician uptake have been broadly described, there has been less work exploring how individuals with acute SI/SA might perceive LMC. Questions remain about how best to promote behavior change (i.e., to reduce home lethal means access) among individuals with acute suicide risk. This is especially true for adults, where it is the at-risk individual (rather than the non-suicidal parent of an at-risk adolescent) who receives LMC and is responsible for making changes. These adults also have unique needs related to understanding of LMC messaging; individuals with active SI/SA being evaluated in an ED are likely to have altered cognition, reasoning, processing, and emotional expression, suggesting the need for tailored messaging, language, and implementation. As provider engagement in LMC increases, the need for tailored communication also increases – tailoring not only with respect to firearms but also to the cognitive state of a suicidal adult.

### Objective

We sought to use qualitative interviews with people with lived experience of SI/SA to explore challenges and strategies related to LMC and effective communication in acute settings such as EDs.

### Study Sample

Participants were a part of a larger study that created a patient-facing decision aid for reducing lethal means access in the context of suicide risk.^14^–^16^ Participants were recruited through direct email invitations, posted flyers, and online advertisements. Eligible participants for the parent project were English-speaking, community-dwelling adults (≥ 18 years) in the United States who did not have active suicidal ideation and who belonged to ≥ 1 stakeholder group: those with “lived experience” of suicide risk (either themselves or a family member); suicide prevention professionals; ED providers; and firearm experts. For this analysis, we included only interviews with adults with “lived experience” of suicide.

## METHODS

One-on-one, semi-structured interviews were conducted between August–December, 2017 via web conference or in person. All interviews lasted approximately 45 minutes and were recorded and professionally transcribed. At the end of the interview, participants completed a questionnaire about their demographic characteristics and received a $25 gift card. All participants provided informed consent and the study was approved by the local institutional review board.

**Population Health Research Capsule**

What do we already know about this issue?

*Lethal means counseling (LMC) is an underutilized resource in emergency department care of adults with suicidal ideations or attempts.*

What was the research question?

*We spoke to those with lived experience of suicidal ideation or attempt to learn how LMC resources could be most beneficial to them.*

What was the major finding of the study?

*Engaging suicidal individuals in LMC may be more effective when messaging and approaches consider their emotional state.*

How does this improve population health?

*By learning from adults with lived experience, we will be better able to design and implement resources to be used by suicidal individuals.*

Interviewers followed a basic guide using broad, open-ended questions to explore decision support needs (i.e., educational needs of adults in crisis and means by which to elicit personal values relevant to decisions about firearm and medication storage) and elicit feedback on iterative versions of the decision aid. Broad interview domains included the following: participants’ prior experiences with decision-making around firearm or medication storage during times of suicide risk; recommendations for decision aid edits (e.g., messaging, formatting, and imagery); and perception of the decision aid’s ability to influence someone being evaluated in an ED for SI/SA (Appendix). A short questionnaire collected demographic information. A professional research assistant with a background in sociology and qualitative research conducted the interviews and conducted primary data analysis. The study team also included Masters- and doctoral-level clinical social workers and physicians with experience in mixed-methods research, emergency medicine, suicidology, crisis intervention, outpatient behavioral health, and shared decision-making. Field notes written during and immediately after the interviews captured nonverbal cues and in-the-moment global understanding of responses.

For analysis, we used a team-based approach informed by established mixed deductive and inductive techniques.^17^–^21^ We used Dedoose analytic software v 7.1.3 (SocioCultural Research Consultants, Los Angeles, CA). Through deductive thematic analysis, we interpreted data in the context of the theoretical framework and existing literature. We combined this with an inductive approach to allow identification of new, emerging themes. Through these techniques, we synthesized codes into a core set of themes, and we compared and contrasted our themes with our first cycle of direct speech coding.^20^ We organized the final core themes into a preliminary framework about conversations related to the suicidal state. Together these processes provided an in-depth, comprehensive analytic matrix for interpretation.^19^,^21^ Our multidisciplinary team provided multiple perspectives through which to interpret the text data, and we shared the themes and framework with participants during the last set of interviews (“member checking”) to further establish thematic organization. Participants were recruited until thematic saturation was reached. We followed the COREQ guidelines for the conduct and reporting of qualitative research projects.^22^

## RESULTS

We conducted 27 interviews with adults who had lived experience of suicide ideation or attempts in either themselves (n = 14) and/or a family member (n = 23; Table 1). Participants had a mean age of 44 and ranged from 25–70 years old. Two-thirds were male (67%) and 89% were White. Eight participants (30%) were firearm owners.

The interviews yielded 450 pages of transcript data and 34 pages of memos. Two dominant themes emerged related to how the affective state of a suicidal person can challenge reasoning and information processing. First, the dominance of emotionality over rationality was seen as a barrier to interventions for an individual in crisis. Second, participants proposed strategies to overcome these challenges through designing interventions with attention to high emotionality. These strategies address three subthemes: initiation; engagement; and context (Table 2).

### Affective State

Participants spoke to the state of mind of individuals with suicidal thoughts or behaviors, including how that state differs from a non-suicidal state. One said, *“When I’m feeling great, I would think I would never grab a firearm and blow my brains out. But when I’m feeling horrible and spiraling down, of course it’s gonna come across my mind.”* When asked about making decisions within this context, interviewees discussed the specific challenges in making decisions posed by the high emotionality of people in crisis. Specifically, they noted LMC tools designed by clinicians and researchers – individuals in rational states – could function poorly for those in a heightened emotional state.

*“When people get into that crisis mode, they’re already overwhelmed. If they’re at the ER or they’re at anywhere, clearly their own resources aren’t working anymore. If you were to tell them, ‘Hey, come up with a plan to keep yourself safe,’ they wouldn’t know what to do. They’d say, ‘That’s why I’m here.’ Versus, ’Pick some things on this list. All of them are good options. Which one’s the best for you?’ I think it can be a lot less taxing.”*

This distinction, as described by a participant, spoke to the need for directed suggestions that guide an individual in making a decision, rather than general counseling about the need to do something without suggested, concrete actions. Another participant elaborated on the importance of providers giving simple steps or clear options to individuals in a suicidal crisis but more detailed information to supporting family or friends (who likely are in a more rational state).

*“‘Wait, so what – is there an answer to this? Like, ‘how do I easily store a weapon if I have one?’ And it was sort of like just – it was almost overwhelming with information. Like I don’t – especially like having been someone who has that sort of crisis mindset, I would look at that and be like, ‘I just don’t know what I’m supposed to do. Can you please just tell me what to do?’ would be sort of how I would have approached it if I were the patient. So I think a simple recommendation, like, ‘You could – here are three ways you can store your guns,’ you know, would be easier than the pros and cons of each of the ways. Although, I think that information could be really valuable for families who are making better decisions and in a better sort of headspace to be able to analyze information; I think that could be helpful.”*

Participants described how too much information can be overwhelming for someone in crisis and emphasized the need for simplicity and identifying someone who can act as support.

### Challenges to Helping an Individual at Risk of Suicide to Make Decisions

#### Initiation

The first challenge identified was how best to initiate discussions with someone in a state of high emotionality (i.e., with acute SI/SA) to discuss lethal means safety and to look at the decision aid. Interviewees discussed that making decisions and digesting information can be difficult, highlighting the need for streamlined graphics and parsimonious text in the decision aid. As one said, *“I wonder if there is a way to do both that doesn’t take up too much space, ‘cause this I think already if you’ve got a person in crisis they’re gonna kind of look at it and go ‘oh my god.’ [Laughs] I think it could be a little overwhelming.”* In sharing this idea, this participant is suggesting the need for clear, simplified information. Supportive messages were also identified as a strategy to encourage connection and initiation of decision aid use (Table 2), including explicit acknowledgement that stress can alter a person’s usual cognitive or decision-making abilities. One participant said: *“You can’t predict that in any person on a normal day, I don’t think, or a group of people on a normal day, and then extrapolating it for each crisis…. I think, you know, ‘when we’re in crisis we’re not quite as we would be otherwise,’ so kind of breaking it down.”* This participant acknowledged that designing and developing resources for any group of people has challenges, and that with high emotionality there is a need for more directness and for accessible language.

#### Ongoing engagement

Once the conversation is initiated, the second challenge identified was how to maintain the attention of the person in the crisis, including how to keep them engaged during LMC and when they return home. Gathering the name and contact information of another individual was suggested as a way to encourage connection to others and maintaining safety-focused changes. The timing of when to encourage individuals in crisis to identify collateral sources of support was also seen as critical.

*“I could see that if somebody just in the moment filling this out, they might be interested in putting in, say, somebody’s email address because they’re in the moment. But as they walk out, they may well think twice about actually reaching out for the help. … They might be in a more vulnerable space in the hospital because they’re probably in the conversation and have been talking about suicidal feelings, which means it sounds to me like it would be an opportunity ripe for being able to send an email to somebody saying ‘[name]’s identified you as the person that he would like to speak to about concerns he had about being safe around his firearms’ or something like that because that would allow my wife or whoever I plug into the thing in the moment to hopefully broach the topic as opposed to relying on me after I get home and cool down a bit.”*

Participants also identified hopeful, supportive language as useful in maintaining user engagement (Table 2), along with simple, discrete choices as described above. This participant talked us through the pieces behind connecting to someone while the person experiencing SI/SA was still in the hospital. The context of the hospital, and conversations that happen during patient care, can be used as a window into continuous care afterward. As one participant said, *“Just telling them that it’s okay to set the guns aside while they’re in crisis, like some reassurance, ‘cause yeah, I guess when you feel like you can’t escape them even if you want to, like what do you do. There’s a sense of helplessness and utility there that we’re trying to avoid.”* Thus, to provide people in crisis with reassurance and encouragement was noted here as helpful in maintaining engagement with resources.

#### Context

The third challenge identified was the context in which the conversation about firearm or medication storage was occurring, including the environment (e.g., ED, hospital, or home) and who else was involved in storage. Participants suggested prompts on how to engage people that they trust in the decision about firearm storage, with a recommendation for a large list of potential support individuals (family, friend, neighbor, fellow veteran, etc) to enable suicidal individuals to choose as many as possible, as well as to prompt them to consider people in their social lives who they may not have thought of during this moment of crisis. A participant who works with veterans commented:

*“Maybe under Friend/Family/Neighbor, you could put ‘another veteran’ or something like that. … The work that we do is you talk to – you can kind of prime the conversation. It would be like, ‘Well, what if your buddy was really struggling? What would you do?’ He was like, ‘I would get in my car and drive 600 miles to go help him out.’ And I said, ‘Well, what would your buddy do for you?’ He was like, ‘I guess they could hold my guns.’”*

The temporary nature of firearm-storage changes for suicide prevention was highlighted as a key concept to reinforce as a way to gain buy-in, encourage behavior change, and reduce the possibility of defensiveness or the feeling that the goal was to undermine lifestyle choice. Recognizing, as this participant did with their friend, the relationships and supports that exist but may have been overlooked before being prompted through comprehensive listing, is again giving a set of options rather than vague, general directions.

## DISCUSSION

Lethal means counseling for those at risk of suicide, including those evaluated in EDs, is important as it may affect home storage behavior and ultimately may reduce suicide risk.^23^,^24^ This qualitative study highlights key considerations about decision-making during a time of crisis. Participants consistently emphasized the overarching needs related to meeting the needs of people in a state of high emotionality, one characterized by high affective valence and lower rationality with attendant cognitive and communicative challenges. The dominant theme was the need for simplification of information being shared with individuals in a state of high emotionality, along with the need to remind them of their desire for connection with others.

This study highlights our understanding of how patients should be able to engage with available resources in a way that positively impacts home safety choices. Lethal means counseling could work in conjunction with ED-based approaches such as safety planning by engaging clients in identifying the treatment and safety plans that are best for them.^25^–^27^ When identifying strategies related to the challenges of initiation and engagement, participants discussed the need for engaging individuals experiencing crisis collaboratively in their own care, including LMC. This is consistent with the collaborative nature of leading treatment approaches for suicidal thoughts and behavior, as well as with shared decision-making.^28^

For example, in dialectical behavior theory (DBT), clients work collaboratively with a social worker or other behavioral healthcare provider to learn skills to help them regulate suicidal thoughts and rapid emotion escalation, with the understanding that different skills are needed in different times and for different purposes, depending on the circumstances, the goals, and emotional state of the patient.^29^ The Collaborative Assessment and Management of Suicidality (CAMS) approach also focuses on collaboration between social workers or other providers and clients in learning to understand the origins of suicidal thoughts, feelings, and behaviors.^30^ The CAMS approach encourages clients to engage in developing their own treatment plan and it can be used within various psychotherapies, including potentially through a virtual interface in EDs.^31^

The type and quality of affective, cognitive, and somatic states among those at highest risk of suicide have been previously documented; they include desperation, hopelessness, rage, abandonment, guilt, anxiety, humiliation, sleep disturbance, avolition, and self-hatred.^32^,^33^ This intense emotional state was also highlighted in our interviews. While most social work, psychology, counseling, divinity, and similar programs offer substantive training in responding to clients experiencing strong emotions, most Masters-trained practitioners (who are typically the behavioral health specialists working in EDs) report feeling inadequately prepared to work with clients during their periods of highest suicide risk.^34^,^35^ These include assistance in reviewing resources and a collaborative approach to identifying concrete next steps. Training resources exist, such as CALM (counseling on access to lethal means) to help support behavioral health and other providers feel confident in engaging in this collaborative LMC working during and after a suicidal crisis.^25^

Overall, the framing that participants felt would be most helpful was addressing the facts in a digestible fashion while still encouraging confidence in the person in crisis. In doing this, participants shared sentiment that reflected the transition between someone in a highly charged emotional state and someone in a typical, more rational, deliberative state, where they could successfully participate in their own care. Seeking and incorporating insight from those who have been in this state of mind can help make approaches such as LMC more accessible to clients, in the same way that CAMS, safety planning, and certain components of DBT are structured to engage clients in their own care.^27^,^29^,^30^

This project lent itself to the understanding of the difficulty inherent in reflecting on being in a “hot state” when one is in a “cold state” – including for the individuals interviewed in this project. The “hot-cold empathy gap”^36^ highlights how it could be possible that reflections and recommendations made by those in a cold state of high rationality might underestimate the volatility of preferences among those in a state of emotionality. While none of our participants identified this dynamic by name, many of them did allude to the labile nature of cognitive processes they either experienced or observed in their loved ones during suicidal crises, and advocated for conservative approaches to communication, facilitation of discussion with healthcare providers, and use of decision support tools.

## LIMITATIONS

Among the limitations of this study was that interviews did not focus solely on the topic discussed here. Thus, although our analysis included 27 individuals, generalizability may be limited. Participation was voluntary with a small incentive, so interviewees may have been particularly passionate about the subject. We did, however, use snowball sampling to contact additional interviewees identified by participants as having unique or influential perspectives. Our interviews did not discuss how intoxication with alcohol or other substances may further affect the cognitive state of an individual with suicide risk. Given the frequent co-occurrence of intoxication and suicidality among ED patients, this is an area that merits further study. Finally, our interviews were in the context of receiving feedback on our specific LMC decision aid. The feedback discussed here is based on broader ideas shared by participants about the considerations needed when communicating with this population of people in crisis.

## CONCLUSION

A key component of care of suicidal individuals in acute care settings – and one that is a policy- and evidence-supported and scalable intervention – is lethal means counseling to reduce access to firearms and other methods of suicide. Incorporating the perspectives of individuals with personal or family-lived experience with suicide can enhance development and delivery of interventions in the ED. Specifically, interventions for those with acute suicide risk should consider the emotional and cognitive states, and needs, of those patients. Directed, digestible information that is supportive, with concrete steps could encourage both collaboration, independence, and engagement in care.

## Supplementary Information



## Figures and Tables

**Table 1:** Characteristics of interview participants (n = 27).

Age (median, IQR, range)	44 (35–50; range 25–70)
Female (n, %)	18 (67%)
Race (≥1 allowed)	
White	24 (89%)
Black	3 (11%)
American Indian/Alaska Native	1 (4%)
Hispanic	5 (19%)
Veteran	3 (11%)
Residence in mostly rural area	5 (19%)
Work in mostly rural area	3 (11%)
Stakeholder group affiliation (≥1 allowed)	
Personal history of suicidal thoughts or attempt	14 (52%)
Family member of someone with suicidal thoughts, attempt, or death	23 (85%)
Firearm owner or enthusiast	8 (30%)
Work at/with firearm retailer, range, or organization	2 (7%)
Work in suicide prevention (including volunteering)	18 (67%)
Healthcare provider	10 (37%)
Work/affiliated with VA or other veteran service provider	4 (15%)

*IQR*, interquartile range; *VA*, Veterans Affairs.

**Table 2:** Representative quotes, by challenges and strategies.

Theme	Challenge	Strategy
Initiation	“I think starting off with something, especially if you are in fact feeling helpless or alone, that starts off with “This tool can help you make a decision,” it sounds like work. [Laughs] And that’s probably the last thing you’re thinking about in that situation.”	“So to my eye the ‘You may feel helpless and alone right now’ probably catches somebody who is feeling helpless and alone and then pulls them in.”
Engagement	“’Preferences, Logistics and Other Issues,’ that sounds pretty cold, really cold, and also kind of technical, that it’s not about a person.”	“So ‘Beliefs and Choices’ or something like that, which is still not too warm and fuzzy, but it’s acknowledging that there’s a human that’s making these decisions.”
Context	“I just don’t think you can hammer the temporary message nearly enough because you think about the history of public health trying to promote safe storage even outside of suicide, like the trigger locks and stuff. … Most of those things didn’t work because people were like, ‘Well, you’re giving me this really clumsy thing, and I gotta find the key, and I have to hide the key or know the combination or whatever. Then I can’t get it when the burglar breaks in.’ So they already have reasons in their head why anything other than immediate access on the nightstand with a chambered gun is a negative thing.”	“So, in hammering home the temporary thing doesn’t make me think, ‘Oh, they’re asking me to change my lifestyle and in terms of how I interact with this firearm. They’re just asking me to keep…’ Even though obviously that’s what we want ideally, but for these things, if we’re talking temporary, just the advertising principle of repetitive messages.”

## References

[1] Mann JJ, Apter A, Bertolote J (2005). Suicide prevention strategies: a systematic review. JAMA.

[2] Miller M, Salhi C, Barber C (2020). Changes in firearm and medication storage practices in homes of youths at risk for suicide: results of the SAFETY Study, a clustered, emergency department-based, multisite, stepped-wedge trial. Ann Emerg Med.

[3] Runyan CW, Becker A, Brandspigel S (2016). Lethal means counseling for parents of youth seeking emergency care for suicidality. West J Emerg Med.

[4] Betz ME, Miller M, Barber C (2016). Lethal means access and assessment among suicidal emergency department patients. Depress Anxiety.

[5] Betz ME, Arias SA, Miller M (2015). Change in emergency department providers’ beliefs and practices after use of new protocols for suicidal patients. Psychiatr Serv.

[6] Diurba S, Johnson RL, Siry BJ (2020). Lethal means assessment and counseling in the emergency department: differences by provider type and personal home firearms. Suicide Life Threat Behav.

[7] Runyan CW, Brooks-Russell A, Tung G (2018). Hospital emergency department lethal means counseling for suicidal patients. Am J Prev Med.

[8] Bulger EM, Kuhls DA, Campbell BT (2019). Proceedings from the Medical Summit on Firearm Injury Prevention: a public health approach to reduce death and disability in the US. J Am Coll Surg.

[9] Pallin R, Spitzer SA, Ranney ML (2019). Preventing firearm-related death and injury. Ann Intern Med.

[10] Barber CW, Miller MJ (2014). Reducing a suicidal person’s access to lethal means of suicide: a research agenda. Am J Prev Med.

[11] Ranney ML, Fletcher J, Alter H (2017). A Consensus-Driven Agenda for Emergency Medicine Firearm Injury Prevention Research. Ann Emerg Med.

[12] Stanley IH, Hom MA, Rogers (2016). Discussing firearm ownership and access as part of suicide risk assessment and prevention: “means safety” versus “means restriction”. Arch Suicide Res.

[13] Marino E, Wolsko C, Keys S (2018). Addressing the cultural challenges of firearm restriction in suicide prevention: a test of public health messaging to protect those at risk. Arch Suicide Res.

[14] Pallin R, Siry B, Azrael D (2019). “Hey, let me hold your guns for a while”: a qualitative study of messaging for firearm suicide prevention. Behav Sci Law.

[15] Betz ME, Knoepke CE, Siry B (2019). ‘Lock to Live’: development of a firearm storage decision aid to enhance lethal means counselling and prevent suicide. Inj Prev.

[16] Betz ME, Knoepke CE, Simpson S (2020). An interactive web-based lethal means safety decision aid for suicidal adults (Lock to Live): pilot randomized controlled trial. J Med Internet Res.

[17] Braun V, Clarke V (2006). Using thematic analysis in psychology. Qual Res in Psychol.

[18] Curry LA, Nembhard IM, Bradley EH (2020). Qualitative and mixed methods provide unique contributions to outcomes research. Circulation.

[19] Fereday J, Muir-Cochrane E (2006). Demonstrating rigor using thematic analysis: A hybrid approach of inductive and deductive coding and theme development. Int J Qual Meth.

[20] Saldana J, Seaman J, Horvai A (2013). First Cycle Coding Methods. The Coding Manual for Qualitative Researchers.

[21] Thomas DR (2003). A general inductive approach for qualitative data analysis.

[22] Tong A, Sainsbury P, Craig J (2007). Consolidated criteria for reporting qualitative research (COREQ): a 32-item checklist for interviews and focus groups. Int J Qual Health C.

[23] Kruesi MJP, Grossman J, Pennington JM (1999). Suicide and violence prevention: parent education in the emergency department. J Am Acad Child Psy.

[24] Roszko PJ, Ameli J, Carter PM (2016). Clinician attitudes, screening practices, and interventions to reduce firearm-related injury. Epidemiol Rev.

[25] Suicide Prevention Resource Center (2020). CALM: Counseling on Access to Lethal Means.

[26] Johnson RM, Frank EM, Ciocca M (2011). Training mental healthcare providers to reduce at-risk patients’ access to lethal means of suicide: evaluation of the CALM Project. Arch Suicide Res.

[27] Stanley B, Brown GK (2012). Safety planning intervention: a brief intervention to mitigate suicide risk. Cogn and Behav Pract.

[28] Barry MJ, Edgman-Levitan S (2012). Shared decision making: the pinnacle of patient-centered care. N Engl J Med.

[29] DeCou CR, Comtois KA, Landes SJ (2019). Dialectical behavior therapy is effective for the treatment of suicidal behavior: a meta-analysis. Behav Ther.

[30] Jobes DA (2012). The Collaborative Assessment and Management of Suicidality (CAMS): an evolving evidence-based clinical approach to suicidal risk. Suicide and Life-Threat.

[31] Dimeff LA, Jobes DA, Chalker SA (2020). A novel engagement of suicidality in the emergency department: virtual collaborative assessment and management of suicidality. Gen Hosp Psychiatry.

[32] Cummins N, Scherer S, Krajewski J (2015). A review of depression and suicide risk assessment using speech analysis. Speech Commun.

[33] Hendin H, Maltsberger JT, Szanto K (2007). The role of intense affective states in signaling a suicide crisis. J Nerv Ment Dis.

[34] Feldman BN, Freedenthal S (2006). Social work education in suicide intervention and prevention: an unmet need?. Suicide and Life-Threat.

[35] Schmitz WM, Allen MH, Feldman BN (2012). Preventing suicide through improved training in suicide risk assessment and care: an American Association of Suicidology Task Force report addressing serious gaps in U.S. mental health training. Suicide and Life-Threat.

[36] Loewenstein G (2005). Hot-cold empathy gaps and medical decision making. Health Psychol.

