# Supplementary material for: Lethal Means Counseling for Suicidal Adults in the Emergency Department: A Qualitative Study
Source: West J Emerg Med. 2021 May 7;22(3):471–7. doi: 10.5811/westjem.2021.8.49485 (PMC8203001; doi:10.5811/westjem.2021.8.49485)
Supplement: Supplementary file 1 [file wjem-22-471-s001.pdf]

Appendix

Interview Guide
Version 1

Research question: What are stakeholder opinions and suggestions about the lethal means decision aid?

Thank you for agreeing to interview with me today. As you know, we are interviewing people for a project to develop a lethal means decision aid to help individuals at risk of suicide make decisions about how to temporarily reduce firearm access. We are speaking with a range of stakeholders, including people with a history of suicide risk (in themselves or family members), healthcare providers, firearm retailers and instructors, and representatives from relevant organizations. We hope to learn from people like you about what might go into this decision and what you think about the draft decision aid. So, please feel free to tell me as much as you can as we go through these questions.

I. Personal Decision Making Experience
  a. First, I would like to better understand what you think influences the decision making process for firearm storage at home.
    1. How did you think people make decisions?
    2. Who helps with those decisions (yours or others')?
    3. Have you ever seen an educational tools, brochure or decision aid to help you in the decision making process?
      a. If so, what were they? How did you feel about them? Did they help you make your decision?
    4. Tell me about your overall decision making process.

II. Paper Decision Aid
  a. In this next part of the interview, I would like to get your feedback about a decision aid we are developing to help future individuals at risk of suicide – along with their family members or friends – decide how to store their firearms to make their home safer in a time of suicide risk. First. I'm going to show you a paper decision aid. Next, I will have you fill out a few short surveys about the decision aid. Then I will ask you some questions about your reaction and opinion of the decision aid. I'd like you to feel free to express your reactions.
  b. [Provide participant copy of decision aid and give them time to review it.]
  c. [Administer Acceptability Questionnaire. Give ample time for completion.]
  d. [Begin interview:]
    1. What is your reaction in general to this decision aid?
      a. Think about your own experience related to decisions like this. How helpful would a tool like this have been for you?
    2. How easy or hard was this decision aid to understand?
      a. Was there anything you didn't understand? What and why?
      b. Are any of the words in this decision aid difficult to understand? Did any seem offensive or off-putting to you?
    3. How balanced was the information about firearm storage options?
      a. Did this decision aid present one option as the best overall choice?
        i. If yes, was there any certain part or parts of the tool that made you feel that way? If yes, was this preference due to the actual evidence in support of one option or due to an unfair presentation of the evidence (or both)?
        ii. Do you have any suggestions to make the decision aid less slanted / more fair and objective?
    4. Some people have told us they learned something new from the decision aid, while others have not. What about you?
    5. What is missing?

a. Are there other questions or important facts that need to be included?

6. What do you like or not like about this decision aid?
7. What do you think of the visuals of the decision aid?
    a. Do you like the layout? The pictures?
8. What advice would you give us to improve it?
9. Is there anything else you think we need to know about this decision aid?

III. Overall Discussion

1. What questions do you have after seeing these decision aids?
2. Some people find that a decision aid may help them figure out what is important to them, that is, what they value. Can you tell me if this decision aid would have helped you to decide what is important to you? If so, how? How could it do a better job at helping with values clarification? (Please give examples)
3. What would you think if your doctor gave or referred you to one of these decision aids?
    a. Would it make you think anything different about your doctor?
4. Do you think any of these decision aids might have changed the way you discussed things with your doctor?
5. Do you think these decision aids would have been helpful for you when you were trying to make a decision?
6. Is there anything else you think we need to know that we haven't discussed?
